# A Simple and High-Throughput Analysis of Amatoxins and Phallotoxins in Human Plasma, Serum and Urine Using UPLC-MS/MS Combined with PRiME HLB μElution Platform

**DOI:** 10.3390/toxins8050128

**Published:** 2016-05-04

**Authors:** Shuo Zhang, Yunfeng Zhao, Haijiao Li, Shuang Zhou, Dawei Chen, Yizhe Zhang, Qunmei Yao, Chengye Sun

**Affiliations:** 1 National Institute of Occupational Health and Poison Control, Chinese Center for Disease Control and Prevention, Beijing 100050, China; zhangshuo6789@sina.com (S.Z.); lihaijiao715@126.com (H.L.); zyz97@263.net (Y.Z.); 2 China National Center for Food Safety Risk Assessment, Key Laboratory of Food Safety Risk Assessment, Ministry of Health, Beijing 100021, China; zhaoyf@cfsa.net.cn (Y.Z.); zhoush@cfsa.net.cn (S.Z.); chendw@cfsa.net.cn (D.C.); 3 The People’s Hospital of Chuxiong Yi Autonomous Prefecture, Chuxiong 675000, China; 18987838279@163.com

**Keywords:** poisonous mushroom, amatoxin, phallotoxin, PRiME HLB, μElution, LC-MS/MS, biological fluids

## Abstract

Amatoxins and phallotoxins are toxic cyclopeptides found in the genus *Amanita* and are among the predominant causes of fatal food poisoning in China. In the treatment of *Amanita* mushroom poisoning, an early and definite diagnosis is necessary for a successful outcome, which has prompted the development of protocols for the fast and confirmatory determination of amatoxins and phallotoxins in human biological fluids. For this purpose, a simple, rapid and sensitive multiresidue UPLC-MS/MS method for the simultaneous determination of α-amanitin, β-amanitin, γ-amanitin, phalloidin (PHD) and phallacidin (PCD) in human plasma, serum and urine was developed and validated. The diluted plasma, serum and urine samples were directly purified with a novel PRiME technique on a 96-well μElution plate platform, which allowed high-throughput sample processing and low reagent consumption. After purification, a UPLC-MS/MS analysis was performed using positive electrospray ionization (ESI+) in multiple reaction monitoring (MRM) mode. This method fulfilled the requirements of a validation test, with good results for the limit of detection (LOD), lower limit of quantification (LLOQ), accuracy, intra- and inter-assay precision, recovery and matrix effects. All of the analytes were confirmed and quantified in authentic plasma, serum and urine samples obtained from cases of poisoning using this method. Using the PRiME μElution technique for quantification reduces labor and time costs and represents a suitable method for routine toxicological and clinical emergency analysis.

## 1. Introduction

Collecting wild mushrooms for food and commercial trade is a traditional hobby in China. However, many edible mushrooms are easily confused with species that are fatally poisonous, which can lead to severe food poisoning [1]. Mushroom poisoning is the main cause of death by food poisoning in China, according to data from the National Management Information System of Public Health Emergencies [2]. Among these toxic mushrooms, the genus *Amanita* is responsible for the most fatalities due to its lethal toxins [1,2], most notably the amatoxins and the phallotoxins, two bicyclic peptide families found in a number of mushrooms [3]. The genera *Galerina*, *Lepiota*, and *Conocybe* are also known to contain amatoxins [4]. Amatoxins, especially α- and β-amanitin, notorious for their high toxicity, interfere with DNA transcription by binding to and inhibiting eukaryotic RNA polymerase II in hepatocytes. Suppressed mRNA synthesis results in cellular necrosis through the inhibition of protein synthesis, and consequently hepatic failure and renal damage can develop, which may lead to death [5]. Although phallotoxins are not considered to have high causative toxicity, it is still necessary to analyze phallotoxins to help identify the mushroom species [6,7].

The initial symptoms of amatoxin poisoning, such as vomiting, abdominal pain, and watery diarrhea, are very difficult to differentiate from the bacterial gastrointestinal disorder and poisoning caused by other non-amanitin-producing mushrooms, such as *Russula*, *Paxillus* and *Boletus*. After the bacterial infection–like period, these symptoms appear to be diminished (12–24 h), but delayed hepatic and renal dysfunctions then occur (24–72 h) [5,8]. Amatoxins are not bound to plasma proteins, and are mostly eliminated through urine. After ingestion [9], they are detectable for approximately 30 h in plasma or serum, and up to 72 h in urine [10]. Therefore, a rapid and confirmatory analysis of amatoxins and phallotoxins in biological fluids is essential for the early diagnosis of mushroom poisoning to avoid confusion with bacterial gastroenteritis or invasive and extensive therapies, including organ transplantation in severe cases.

Although several methods have been described to determine amatoxins and/or phallotoxins in biological fluids, including radioimmunoassay (RIA) [11,12,13], enzyme-linked immunosorbent assay (ELISA) [14,15], capillary zone electrophoresis (CZE) [16,17], and liquid chromatography (LC) combined with ultraviolet (UV) [7,18,19] or electrochemical detection (ECD) [20,21,22], various problems, such as false positives, unstable reproducibility, poor confirmatory ability and laborious procedures, have limited their application in practice [19,23]. Methods combining LC with mass spectrometry (MS), such as triple quadrupole tandem MS (MS/MS) [24,25,26,27,28], time-of-flight (TOF) MS [29] and matrix-assisted laser desorption ionization time-of-flight MS (MALDI-TOF MS) [30], have been reported for the detection of amatoxins and phallotoxins. LC-MS/MS is a powerful technique allowing high sensitivity, reproducibility and specificity, and is less expensive than high-resolution MS. Therefore, we developed a rapid, convenient high-throughput method for early diagnosis to determine the toxins qualitatively and quantitatively and to avoid internal standards (some of which are commercially unavailable) and expensive high-resolution instrumentation.

Although solid-phase extraction (SPE) is a well-known technique for the effective purification of the complex matrix, most of the available SPE methods require laborious conditioning, evaporation, and reconstitution steps. For blood samples, especially plasma, extra protein precipitation before the clean-up step is necessary. To overcome these drawbacks of the traditional SPE method, the OASIS PRiME HLB μElution 96-well plate (Waters, Milford, MA, USA), a novel micro-SPE platform, was introduced to minimize the time and reagent costs. In this 96-well plate only 2 mg of sorbents are loaded in each well, thus substantially reducing the amount of sample, solvent and generated waste during the procedure. We took advantage of its excellent ability to remove phospholipids from biological samples to reduce matrix effects. The present study is a validation of this method for the quantification of α-amanitin, β-amanitin, γ-amanitin, phalloidin (PHD) and phallacidin (PCD) in human plasma, serum and urine using LC-MS/MS. It is also the first study to use the PRiME HLB μElution platform for sample preparation in amatoxin and phallotoxin analysis, which could become the most practical method for routine toxicological and clinical purposes.

## 2. Results and Discussion

### 2.1. Optimization of LC-MS/MS

Mass spectrometric parameters were initially optimized by full scan and daughter scan under positive and negative modes for each analyte using infusion combined with LC. The [M + H]^+^ ion was chosen as the precursor ion for all analytes. Table 1 lists the characteristic ions and collision energy for each compound during multiple reaction monitoring (MRM) acquisition. The proposed structures of the corresponding product ions are presented in the Supplementary Materials section (Figure S1).

For liquid chromatographic separation, several UPLC columns were tested, including ACQUITY UPLC HSS T3 (2.1 mm × 100 mm, 1.8 μm), ACQUITY UPLC BEH C18 (2.1 mm × 100 mm, 1.7 μm) and CORTECS UPLC C18+ (2.1mm × 100 mm, 1.6μm) separation columns under their optimal elution conditions. Since α-amanitin (*m/z* 919.5 > 86.0) and β-amanitin (*m/z* 920.5 > 86.0) both have high carbon contents and their molecular weights differ by only 1 Da, with a ^13^C abundance of 42%–43%, the isotopic substituted α-amanitin (*m/z* 920.5 > 86.0) could interfere with the detection of β-amanitin (*m/z* 920.5 > 86.0). Therefore, the analytes must be completely separated in LC to avoid interference from each other. The CORTECS UPLC C18+ column resulted in an increase in both the retention and resolution of α- and β-amanitin, with sharper peak shapes and stronger MS responses.

### 2.2. Sample Preparation

Acetonitrile and methanol were used as common extractants, but the extraction efficiency proved to be unacceptably low in all types of matrices (recoveries were below 50%). The peptide analytes may have co-precipitated with proteins from the matrix. Moreover, the solvent had to be changed to fit the initial polarity of the following SPE purification. Thus, direct sample dilution is preferred over protein precipitation before the purification step.

**Figure 1:**
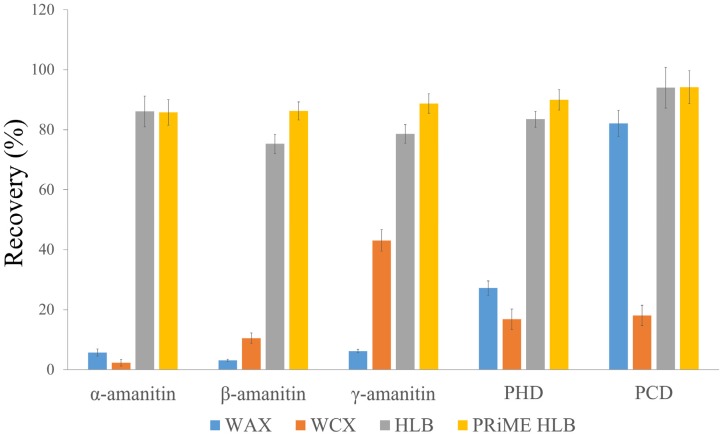
Effects of the different sorbents on the extraction efficiency (recoveries %) (*n* = 3) of 10 ng/mL α-amanitin, β-amanitin, γ-amanitin, PHD and PCD in methanol-water–dissolved standard mixture.

SPE is an important technique for biological sample purification. Several SPE cartridges, principally based on two different mechanisms of retention, have been applied to amatoxin analysis [19,23,25,26,27]. To find the most suitable purification mechanism for all analytes and recovery differences, four types of SPE cartridge were screened in the present study. Based on the ion exchange mechanism, considering the amphoterism of the peptide analytes, a weak anion exchange SPE cartridge (Oasis WAX, 30 mg, 1 cc) and a weak cation exchange SPE cartridge (Oasis WCX, 30 mg, 1 cc) were tested. For the reversed phase mechanism, HLB and PRiME HLB cartridges were tested. The detailed extraction procedures for WAX, WCX, HLB and PRiME HLB cartridges are shown in the Supplementary Materials. The extraction efficiency was determined using a 10 ng/mL aqueous standard mixture (Figure 1). For WAX and WCX, satisfactory recoveries could not be obtained for all analytes. Although all these compounds are characterized as bicyclopeptides containing an unnatural tryptophan residue, they bear different substituent groups [31]. The presence or absence of OH, NH_2_, CH_3_, and COOH makes their acidic-basic properties diverse, so their separation behaviors can be quite different on an ion exchange mode SPE cartridge. HLB and PRiME HLB cartridges showed good recoveries (75.3%–94.2%) for all analytes. To seek an easier and faster method for routine analysis for a large number of samples, we applied a method using a 96-well μElution plate. Compared with common procedures, this preparation method substantially reduces the amount of sample, solvent and generated waste during the procedure, due to the high concentrating ability of the sorbent particles. Additionally, the eluent in the wells was directly diluted for injection in our method, which avoids the time-consuming steps of solvent evaporation or solvent exchange under N_2_ in other methods.

**Figure 2:**
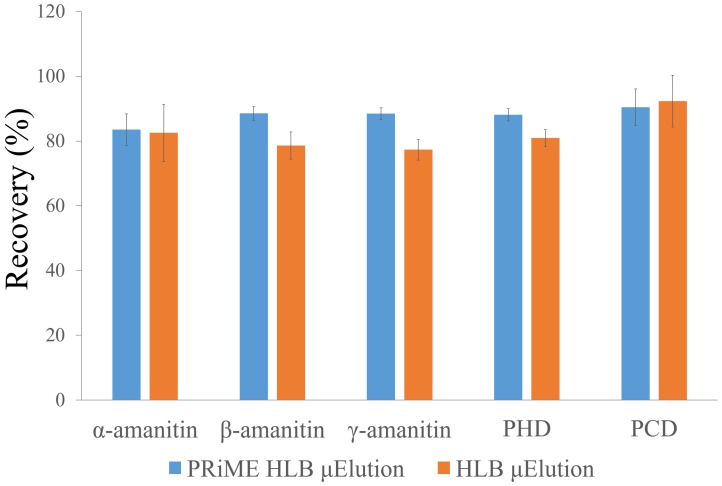
Comparison of extraction efficiency (recoveries %) of HLB and PRiME HLB μElution for 10 ng/mL α-amanitin, β-amanitin, γ-amanitin, PHD and PCD in blank plasma-spiked sample (*n* = 3).

HLB and PRiME HLB mechanisms were chosen for further optimization on the μElution platform. To compare the performance of the two μElution extraction methods, blank samples spiked with 10 ng/mL plasma were prepared. The HLB μElution and PRiME HLB μElution protocols are shown in the Supplementary Materials. Better recoveries and coefficient of variances (CVs) were observed for PRiME HLB μElution (Figure 2).

**Figure 3:**
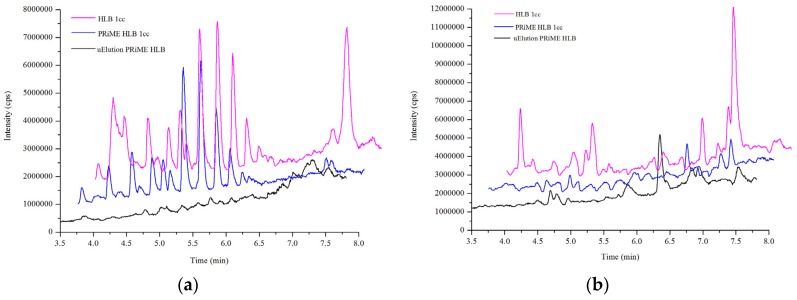
Comparison of LC/MS/MS chromatograms for IS-MRM transition for (**a**) GPChos, *m/z* 184 > 184; (**b**) Lyso-GPChos, *m/z* 104 > 104 in plasma extracts, at 90 V cone voltage and 7 V collision energy.

The matrix effect of biological samples is mainly caused by endogenous components, such as carbohydrates, mineral salts, fats and other metabolites, especially phospholipids. Phospholipids structurally contain phosphate head groups with negative charges, while their quarternary amines confer a positive charge, which is known to cause significant LC-MS/MS matrix ionization effects [32,33]. Although applying matrix-matched calibration curves can partly compensate for signal suppression from the matrix effect, the sensitivity is also compromised. To compare the phospholipid clean-up efficiency of the HLB, PRiME HLB and PRiME HLB μElution techniques, characteristic ions with *m/z* 184.0 > 184.0 and *m/z* 104.0 > 104.0 formed from glycerophosphocholines (GPChos) and lysoglycerophosphocholines (Lyso-GPChos) in plasma extracts, respectively, were obtained after the clean-up procedures were monitored via in-source multiple reaction monitoring (IS MRM) [34]. Comparing the responses of extracts from the same pooled plasma, the PRiME-based method removed more than 90% of GPChos in the matrix, while PRiME HLB μElution showed the best adsorptivity for the selective clean-up of Lyso-GPChos (Figure 3).

Under all the optimized conditions, the pretreatment procedure in the PRiME HLB μElution method proved efficient. In our method, the matrix interference was well controlled and satisfactory recoveries were obtained. Furthermore, the preconditioning step was found unnecessary, and the evaporation step was replaced by simultaneous concentration with elution. Thus, the loss of analytes caused by evaporation was eliminated. As a result, the time required for the extraction and clean-up of 96 samples totaled approximately 15 min, far quicker than the cartridge method. The reagent cost was also lower, and the volume of waste decreased from 2 mL to 600 μL for each sample.

### 2.3. Method Validation

Selectivity, accuracy, precision, matrix effects, linearity, and stability were validated for this method.

#### 2.3.1. Specificity/Selectivity

**Figure 4:**
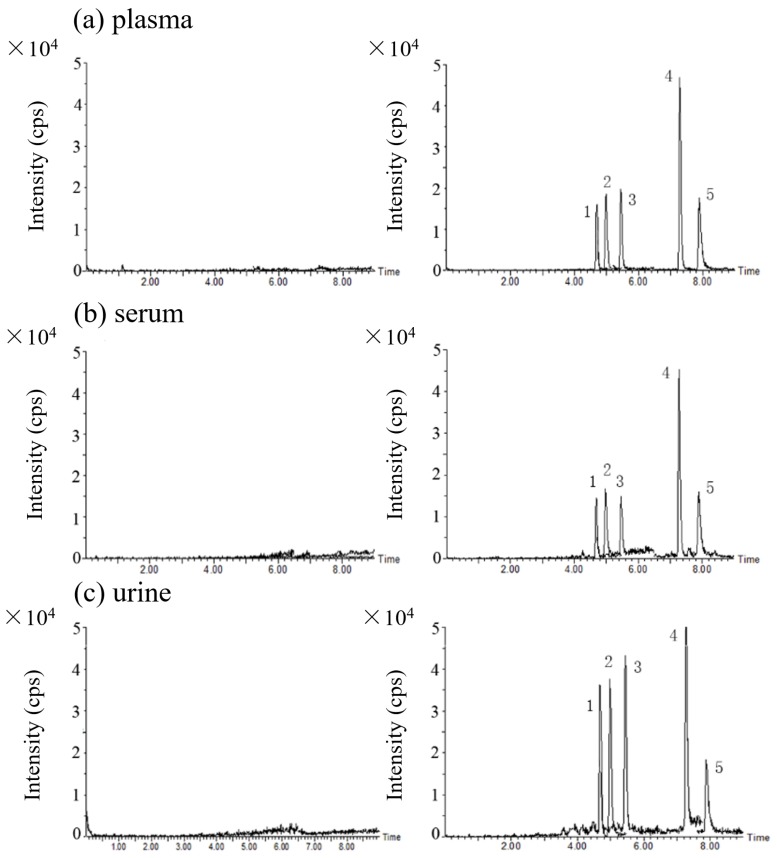
The LC-MS/MS MRM chromatograms of blank matrices (left) and (1) α-amanitin, (2) β-amanitin, (3) γ-amanitin, (4) PHD and (5) PCD at their LLOQ levels in (**a**) plasma, (**b**) serum and (**c**) urine (right), respectively. The five quantification ion transitions (*m/z* 919.5 > 86.0, *m/z* 920.5 > 86.0, *m/z* 903.0 > 86.0, *m/z* 789.4 > 157.0, *m/z* 847.0 > 157.0) are overlapped in the chromatograms.

Twenty blank urine samples were taken from healthy volunteers and treated with the SPE procedure and LC-MS/MS method described in Materials and Methods section (section 3). The samples were then analyzed and compared with the corresponding spiked urine samples at the lower limit of quantification (LLOQ) level to check for possible interference with the detection of the analytes. Twenty samples of blank plasma and 20 samples of blank serum were equivalently analyzed for the same purpose. Representative chromatograms are shown in Figure 4. No obvious interfering peak from blank samples was detected.

#### 2.3.2. Recovery, Matrix Effects, and Process Efficiency

Three types of standard curves were set up to determine the absolute recoveries (RE), matrix effect (ME) and process efficiency (PE). Set I curves were standard solution curves, plotted using water-dissolved standard solutions of 0.2, 0.5, 1, 2, 5, 10 and 20 ng/mL. The Set II curves were matrix-matched standard curves obtained by combining aliquots of working solutions followed by dilution with extracts of blank samples at concentrations of 1, 2, 5, 10, 20, 50 and 100 ng/mL. They also served as calibration curves. Finally, the Set III curves were obtained from matrix-fortified standard samples, plotted by using extracts of blank pooled samples spiked before sample preparation at levels of 1, 2, 5, 10, 20, 50 and 100 ng/mL and serving as quality control (QC) samples. The ME, RE, and PE values were calculated with Matuszewski’s algorithm [35]: ME was determined as the percentage ratio between the slope of Set II and the slope of Set I. RE was calculated as the percentage ratio of the slope of Set III to the slope of Set II. Finally, PE was obtained by multiplying the percentage ratio between the slopes of Set III and Set I by 100. In our research, the ME, representing the relative matrix effect [36], was studied to assess the interference from the sample matrix and the individual differences among biological fluids originating from different subjects or at various time points. We calculated the precision of the slopes of Set II standard lines, which were constructed for six different lots of urine, serum and plasma samples. The value of the matrix effect should be within 80%–120% and the CV of the relative matrix effects should be within 15% [37]. RE, ME, and PE results with their CVs are shown in Table 2. The REs were 80.69% or more for amatoxins and 86.33% or more for phallotoxins. The intra-day precision ranged from 2.14% to 7.01%, and the inter-day precision from 2.42% to 8.54%. The signals of all analytes were matrix-enhanced or suppressed in various degrees and the CVs of the MEs calculated from the six lots of matrices were 6.80% or less.

#### 2.3.3. Linearity/Work Range

The matrix-matched calibration curves, *i.e.*, the Set II standard curves, were prepared as mentioned above, over a linear range from the LLOQ of each analyte at seven concentrations between 0 and 100 ng/mL. The α-, β-, γ-Amanitin, PHD and PCD spiked in blank sample extracts at concentrations of 1, 2, 5, 10, 25, 50, 75 and 100 ng/mL were analyzed. One calibration curve was analyzed each day for three days. Linearity was evaluated using the least squares regression algorithm with a weighting factor of 1/concentration. Correlation coefficients were obtained and residual analysis was performed to test the linear model. All calibration curves gave good linearities for plasma, serum and urine, with correlation coefficient (*r*^2^) values greater than 0.998 (Table 3).

#### 2.3.4. Limit of Detection and Lower Limit of Quantification

The limit of detection (LOD) and LLOQ were obtained from the intensity of 20 blank pooled urine, serum and plasma samples, respectively (Table 3). The LOD was determined as signal:noise ratio > 3:1 for the chromatographic response. The LLOQ was determined as signal:noise ratio > 10:1. The LOD and LLOQ obtained in the present study are at similar or lower levels compared with previous LC-MS/MS reports, but cover five compounds including both amatoxins and phallotoxins. Previous research into the kinetics of amatoxin poisoning in humans has been limited, while the kinetic data for phallotoxins remain unknown. According to Jaeger [9], plasma amatoxins were detected at 8–90 ng/mL and 23.5–162 ng/mL for α- and β-amanitin, respectively. In urine, amatoxin concentrations were 48–4820 ng/mL and 75–7103 ng/mL for α- and β-amanitin, respectively. Therefore, the present method is sufficiently sensitive to detect amanitins in urine and blood in cases of mushroom poisoning in humans.

#### 2.3.5. Stability

The stability of the analytes in the studied matrices was evaluated using the Set III samples (blank matrix spiked with analytes before extraction at levels of 1, 2, 5, 10, 20, 50, 75 and 100 ng/mL) at different storage time periods (after one, two, four and eight weeks). The results of the stability evaluation are shown in Table 4. The recoveries (75.15%–100.86%) of the analytes in plasma, serum and urine showed no significant changes as a function of storage time, with precisions ranging from 1.98% to 11.99%.

## 3. Materials and Methods

### 3.1. Reagents and Materials

The α-, β-, γ-Amanitin and PHD (≥90% purity) were purchased from Enzo Life Sciences (Farmingdale, NY, USA); PCD (≥85% purity) was purchased from Sigma-Aldrich (St. Louis, MO, USA). Ultra-pure water was obtained from Millipore System (Molsheim, France). Ammonium acetate and formic acid were obtained from Merck (Darmstadt, Germany); acetonitrile and methanol were obtained, respectively, from JT Baker (Deventer, Holland) and Fisher Chemical (Leicestershire, UK). Oasis HLB 1 cc (30 mg), Oasis WAX 1 cc (30 mg), Oasis WCX 1 cc (30 mg), and Oasis HLB 1 cc (30 mg) cartridges, as well as Oasis HLB μElution and Oasis PRiME HLB μElution plates, were purchased from Waters (Milford, MA, USA); ACQUITY UPLC HSS T3 (2.1 mm × 100 mm, 1.8 μm), ACQUITY UPLC BEH C18 (2.1 mm × 100 mm, 1.7 μm) and CORTECS UPLC C18+ (2.1 mm × 100 mm, 1.6 μm) separation columns were purchased from Waters (Milford, MA, USA).

Human blank plasma, serum, and urine samples were obtained from healthy volunteers. The authentic biological samples were collected from 31 patients of suspected mushroom poisoning in the People’s Hospital of Chuxiong Yi Autonomous Prefecture. All patients gave their informed consent for inclusion before they participated in the study. The study was conducted in accordance with the Declaration of Helsinki, and the protocol was approved by the Ethics Committee of The 307th Hospital of Chinese People’s Liberation Army (Project identification code 20140354181939) in 25 February 2014.

### 3.2. Preparation of Calibration Standards

Individual stock standard solutions of α-amanitin, β-amanitin, γ-amanitin, PHD and PCD were prepared in methanol (1.0 mg/mL) and stored in the dark at −20 °C. Standard working solutions of each analyte ranging from 100 ng/mL to 1 μg/L were prepared by serial dilution of the stock solutions with ultra-pure water and stored at 4 °C.

Three types of standard curve were prepared in our research. Set I curves were standard solution curves, plotted by using water-dissolved standard solutions of 0.2, 0.5, 1, 2, 5, 10, 15 and 20 ng/mL. The Set II curves were matrix-matched standard curves obtained by combining aliquots of working solutions followed by dilution with extracts of blank samples at concentrations of 1, 2, 5, 10, 20, 50, 75 and 100 ng/mL, which also served as calibration curves. Finally, the Set III curves were obtained from matrix-fortified standard samples, plotted by using extracts of blank pooled samples spiked before sample preparation at levels of 1, 2, 5, 10, 20, 50, 75 and 100 ng/mL and serving as QC samples.

### 3.3. Sample Preparation

#### 3.3.1. Urine Samples

The mixture of 100 μL urine sample and 100 μL ammonium acetate buffer (1M, pH 5) was slowly loaded onto the PRiME HLB μElution 96-well plate. Each well was washed with 200 μL of 5% methanol in water (*v/v*) twice. The compounds were eluted with 25 μL of 95% methanol in water (*v/v*) twice, collected in a clean 96-well collection plate, and subsequently diluted with 450 μL of mobile phase solution (0.2% formic acid in distilled water) prior to LC-MS/MS analysis.

#### 3.3.2. Plasma and Serum Samples

The mixture of 100 μL plasma or serum sample and 100 μL of 4% H_3_PO_4_ in distilled water was slowly loaded onto the PRiME HLB μElution 96-well plate. Then each well was washed with 200 μL of 5% methanol in water twice. The retained compounds were eluted into a clean 96-well collection plate with 25 μL 95% methanol in water twice. The eluent was simply diluted with 450 μL of the mobile phase solution before LC-MS/MS analysis.

### 3.4. LC-MS/MS Analysis

The analytes were identified and quantified on a xevo TQ MS detector (Waters, Milford, MA, USA) coupled to the UPLC ACQUILITY system (Waters, Milford, MA, USA). In the chromatographic system, a CORTEX UPLC C18+ (2.1 mm × 100 mm, 1.6 μm) column was used for separation. The mobile phases were 0.2% formic acid in water (mobile phase A) and 0.2% formic acid in methanol (mobile phase B). A gradient program was started with 95% A and 5% B, with phase B increasing linearly to 30% in the first 4 min, linearly to 40% at 6 min and remaining constant for 2 min. The mobile phase then returned to the initial composition and equilibrated for 2 min prior to the next injection. The total run time for each sample was 10 min. The flow rate was 0.2 mL/min, and the injection volume was 5 μL. The column temperature was 60 °C.

MS/MS detection was carried out on a Waters xevo TQ-S mass spectrometer. MRM was used to measure the target analytes with the positive electrospray ionization (ESI+) mode. The MS conditions were as follows: capillary voltage, 3.0 kV; desolvation gas (600 L/h, 500 °C), nitrogen; collision gas (0.15 mL/min), argon. The MRM parameters for each compound and ion transition were optimized, as summarized in Table 1.

## 4. Method Application

**Figure 5:**
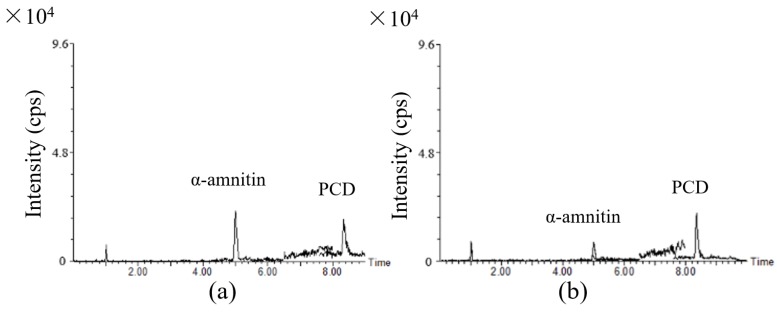
The overlapped LC-MS/MS MRM chromatograms of positive urine samples (**a**) α-amanitin at 6.02 ng/mL and PCD at 2.24 ng/mL; (**b**) α-amanitin at 1.98 ng/mL and PCD at 2.52 ng/mL.

Authentic samples from 31 patients with acute accidental poisoning with wild mushrooms were collected from the hospital at 48–192 h post-ingestion, mostly after 72 h, including 24 urine samples, 30 plasma samples and three serum samples. They were analyzed using the present method. The α-Amanitin and PCD were found in two samples of urine. For one sample, α-amanitin and PCD were at 6.02 and 2.24 ng/mL respectively, and for the other, they were at 1.98 and 2.52 ng/mL, respectively (the chromatograms are shown in Figure 5). However, none of the target toxins were detected in plasma or serum. Detailed urine sample information is given in the Supplementary Materials. This result is consistent with previous studies showing that amanitins can be eliminated very rapidly from blood within 30 h after ingestion, but can remain for three days in urine [9]. Besides, only a few of the wild mushrooms the patients consumed were obtained and identified as the genus *Amanita*. The other possible explanation for the low positive rate is that the species of the wild mushrooms in most cases is unknown, and they probably are not amatoxin- or phallotoxin-producing species, but they may also cause gastrointestinal (GI) symptoms and increased AST or ALT levels, such as the genus *Russula* and *Boletus* do. In the positive cases, the patients presented with early GI symptoms and elevated liver enzymes. The ingested mushrooms were also collected and identified morphologically and phylogenetically as *Amanita exitialis*, a poisonous mushroom that has only been reported in China, and contains α, β-amanitin and PCD among the five target toxins [38].

## 5. Conclusions

A rapid, convenient and high-throughput LC-MS/MS method was developed for the simultaneous analysis of α-amanitin, β-amanitin, γ-amanitin, PHD and PCD in urine, plasma and serum using the PRiME μElution technique for labor-reducing and time-saving purposes. The method has been fully validated, and was successfully applied to authentic biological fluid sample analysis. This method is faster, more convenient and more cost-effective than existing methods; it also avoids internal standards and expensive instrumentation. In summary, this method is suitable for the routine emergency toxicological and clinical determination of amatoxins and phallotoxins in human biological fluids.

## Figures and Tables

**Table 1:** MRM parameters and retention times for target compounds.

Compound	MRM Transition	Cone Voltage (V)	Collision Energy (eV)	Retention Times (min)	Ion Abundant Ratio (% of Base Peak)
α-amanitin	919.5 > 86.0 ^1,2^	20	68	4.72 ± 0.2	38 ± 10
	919.5 > 259.1 ^1^		42		
β-amanitin	920.5 > 86.0 ^1,2^	20	71	4.96 ± 0.2	47 ± 9
	920.5 > 259.1 ^1^		42		
γ-amanitin	903.0 > 86.0 ^1,2^	20	70	5.45 ± 0.2	43 ± 8
	903.0 > 243.1 ^1^		41		
PHD	789.4 > 157.0 ^1,2^	20	61	7.31 ± 0.2	91 ± 4
	789.4 > 86.0 ^1^		70		
PCD	847.0 > 157.0 ^1,2^	20	64	7.87 ± 0.2	83 ± 5
	847.0 > 86.0 ^1^		70		

^1^ the confirmation ion transitions; ^2^ the quantification ion transitions.

**Table 2:** Recovery, intra-day and inter-day precision values for amatoxins and phallotoxins in human plasma, serum and urine.

Compound	Matrix	Relative Matrix Effect % (CV%) *n* = 6	Process Efficiency %	Recovery % *n* = 6	Precision % *n* = 6		LOD ng/g *n* = 20	LLOQ ng/g *n* = 20
					Intra-day	Inter-day		
α-amanitin	plasma	105.18 (3.05)	87.84	83.51	5.86	6.68	0.5	1
	serum	98.25 (6.52)	80.93	82.37	4.76	5.03	0.5	1
	urine	91.92 (6.80)	77.43	84.24	3.94	4.02	1	2.5
β-amanitin	plasma	103.37 (4.44)	91.56	88.58	5.43	2.42	0.5	1
	serum	100.48 (3.08)	85.50	85.10	7.01	5.46	0.5	1
	urine	96.80 (3.85)	78.11	80.69	3.68	3.22	0.5	1
γ-amanitin	plasma	98.51 (4.82)	87.17	88.49	3.01	2.57	0.5	1
	serum	105.47 (3.97)	95.11	90.18	2.14	4.04	1	2.5
	urine	105.99 (4.11)	87.62	82.67	3.29	5.67	1	2.5
PHD	plasma	103.83 (3.44)	91.49	88.12	2.16	5.53	0.5	1
	serum	103.57 (3.39)	96.21	92.90	3.92	5.71	0.5	1
	urine	103.84 (3.36)	91.01	87.65	3.44	4.03	0.5	1
PCD	plasma	99.03 (3.17)	99.46	90.44	6.21	8.54	0.5	1
	serum	105.77 (2.54)	91.79	86.78	2.72	3.04	0.5	1
	urine	94.58 (3.62)	81.65	86.33	4.09	7.22	0.5	1

**Table 3:** The slopes of regression equations and correlation coefficients of target compounds in a different matrix.

Compound	Slopes of Set I (CV%), Correlation Coefficient (*r*^2^) (*n* = 6)	Matrix	Slopes of Set II (CV%), Correlation Coefficient (*r*^2^) (*n* = 6)	Slopes of Set III (CV%), Correlation Coefficient (*r*^2^) (*n* = 6)
α-amanitin	1306.37 (0.84), 0.9993	plasma	1374.01 (3.05), 0.9996	1243.57 (5.86),0.9998
		serum	1294.35 (6.52), 0.9993	1011.68 (4.76), 0.9989
		urine	1200.82 (6.80), 0.9998	1061.60 (3.94), 0.9998
β-amanitin	1534.87 (1.03), 0.9998	plasma	1510.67 (4.44), 9.9998	1338.17 (2.43), 0.9994
		serum	1542.29 (3.08), 0.9983	1306.25 (6.01), 0.9978
		urine	1485.85 (3.85), 0.9987	1235.27 (2.68), 0.9995
γ-amanitin	952.75 (0.96), 0.9996	plasma	938.58 (4.82), 0.9998	933.87 (2.01), 0.9992
		serum	1004.89 (3.97), 0.9986	875.13 (2.14), 0.9967
		urine	1009.82 (4.11), 0.9999	906.19 (3.29), 0.9998
PHD	3434.45(0.95), 0.9998	plasma	3595.76 (3.44), 0.9998	3600.11 (2.16), 0.9976
		serum	3557.01 (3.39), 0.9997	3125.76 (3.92), 0.9994
		urine	3566.29 (3.36), 0.9998	3304.45 (3.44), 0.9993
PCD	1442.27 (0.99), 0.9998	plasma	1428.30 (3.17), 0.9999	1434.52 (6.21), 0.9992
		serum	1525.57 (2.54), 0.9997	1177.65 (2.72), 0.9987
		urine	1364.07 (3.62), 0.9998	1339.23 (4.09), 0.9983

**Table 4:** Stability data for amatoxins and phallotoxins in human plasma, serum and urine (*n* = 3).

Compound	Matrix	One Week	Two Weeks	Four Weeks	Eight Weeks
		Recovery% (Precision%)			
α-amanitin	plasma	87.12 (5.52)	90.95 (3.52)	94.56 (7.23)	93.73 (7.31)
	serum	83.31 (4.57)	87.25 (8.74)	85.15 (6.79)	84.42 (10.04)
	urine	81.03 (3.25)	86.32 (5.54)	83.64 (4.54)	80.43 (3.40)
β-amanitin	plasma	82.56 (5.72)	80.77 (4.72)	84.78 (8.79)	83.38 (9.09)
	serum	75.15 (2.98)	83.26 (5.08)	80.63 (5.08)	87.55 (4.99)
	urine	87.54 (3.76)	83.94 (3.60)	85.42 (2.65)	89.12 (2.95)
γ-amanitin	plasma	91.33 (1.98)	96.68 (11.99)	94.44 (3.90)	96.61 (8.30)
	serum	85.27 (2.88)	88.71 (4.58)	86.04 (6.82)	83.14 (3.82)
	urine	78.34 (3.25	83.42 (6.94)	85.23 (5.43)	76.23 (5.43)
PHD	plasma	95.87 (3.12)	98.66 (3.22)	97.42 (6.26)	93.40 (6.27)
	serum	90.03 (7.38)	97.04 (3.28)	95.11 (5.24)	100.86 (6.72)
	urine	80.62 (6.10)	80.62 (5.05)	85.24 (9.05)	90.50 (7.55)
PCD	plasma	96.41 (4.34)	90.65 (3.36)	92.18 (2.35)	88.35 (4.56)
	serum	85.24 (3.66)	83.49 (5.16)	82.55 (4.36)	87.52 (6.01)
	urine	87.55 (5.45)	84.56 (7.91)	86.49 (6.88)	83.92 (3.85)

## Supplementary Materials

The following are available online at www.mdpi.com/2072-6651/8/5/128/s1. Figure S1: The proposed fragmentation of the analytes by MS/MS mode to their products, Table S1: The information of 24 urine samples with mushroom poisoning.
