# Supplementary material for: A Simple and High-Throughput Analysis of Amatoxins and Phallotoxins in Human Plasma, Serum and Urine Using UPLC-MS/MS Combined with PRiME HLB μElution Platform
Source: Toxins (Basel). 2016 May 4;8(5):128. doi: 10.3390/toxins8050128 (PMC4885043; doi:10.3390/toxins8050128)
Supplement: Supplementary file 1 [file toxins-08-00128-s001.pdf]

*Toxins* **2016**, *8*, 128; doi:10.3390/toxins8050128 

# Supplementary Materials: A Simple and High-Throughput Analysis of Amatoxins and Phallotoxins in Human Plasma, Serum and Urine Using UPLC-MS/MS Combined with PRiME HLB μElution

**Shuo Zhang, Yunfeng Zhao, Haijiao Li, Shuang Zhou, Dawei Chen, Yizhe Zhang, Qunmei Yao and Chengye Sun**

The extraction procedures for WAX, WCX, HLB and PRiME HLB cartridges in detail:

WAX: a WAX 1cc/30 mg cartridge was preconditioned with methanol and water, then 1mL 10 ng/mL water-dissolved standard mixture was loaded onto the cartridge. After washing the cartridge with 1 mL 2% formic acid in water and 1 mL methanol, the analytes were eluted with 1 mL 5% $NH_3 \cdot H_2O$ in methanol. The eluent was evaporated under $N_2$ and the residues were diluted with initial mobile phase.

WCX: a WCX 1cc/30 mg cartridge was preconditioned with methanol and water, then 1 mL 10 ng/mL water-dissolved standard mixture was loaded onto the cartridge. After washing the cartridge with 1 mL 2% $NH_3 \cdot H_2O$ in water and 1 mL methanol, the analytes were eluted with 1 mL 2% formic acid in methanol. The eluent was evaporated under $N_2$ and the residues were diluted with initial mobile phase.

HLB: a HLB 1cc/30 mg cartridge was preconditioned with methanol and water, then 1 mL 10 ng/mL water-dissolved standard mixture was loaded onto the cartridge. After washing the cartridge with 1 mL 5% methanol in water, the analytes were eluted with 1 mL methanol. The eluent was evaporated under $N_2$ and the residues were diluted with initial mobile phase.

PRiME HLB: 1 mL 10 ng/mL water-dissolved standard mixture was loaded onto the PRiME HLB cartridge. After washing the cartridge with 1 mL 5% methanol in water, the analytes were eluted with 1 mL methanol. The eluent was evaporated under $N_2$ and the residues were diluted with initial mobile phase.

The HLB μElution and PRiME HLB μElution protocols:

HLB μElution: afer conditioning the HLB μElution 96-well plate with 200 μL of MeOH and water, 100 μL of 10 ng/mL plasma QC sample and 100 μL 4% $H_3PO_4$-water solution mixture was transferred onto the well. The loaded plate was then washed with 200 μL of 5% methanol in water twice and the sample was eluted with 25 μL of methanol–water (95/5, *v*/*v*) twice. The eluent was diluted with 450 μL initial mobile phase.

PRiME HLB μElution: 100 μL of 10 ng/mL plasma QC sample and 100 μL 4% $H_3PO_4$-water solution mixture was transferred onto the PRiME HLB μElution 96-well plate. The loaded plate was then washed with 200 μL of 5% methanol in water twice and the sample was eluted with 25 μL of methanol-water (95/5, *v*/*v*) twice. The eluent was diluted with 450 μL initial mobile phase.

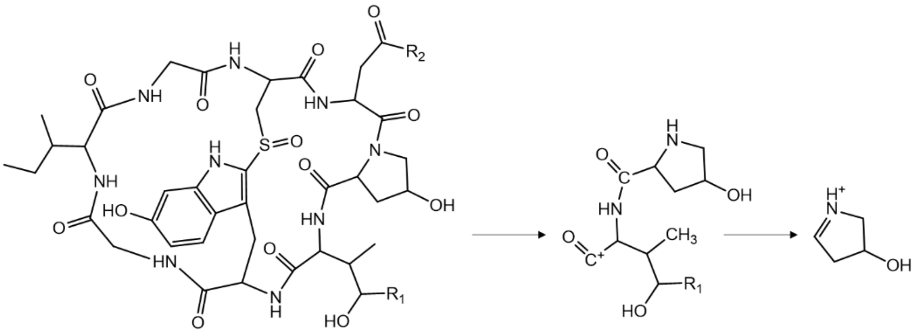

α-amanitin: $R_1$ = $CH_2OH$, $R_2$ = $NH_2$, Mw: 918.97 m/z: 259.13 m/z: 86.06
β-amanitin: $R_1$ = $CH_2OH$, $R_2$ = OH, Mw: 919.95 m/z: 259.13 m/z: 86.06
γ-amanitin: $R_1$ = $CH_3$, $R_2$ = $NH_2$, Mw: 903.97 m/z: 243.13 m/z: 86.06

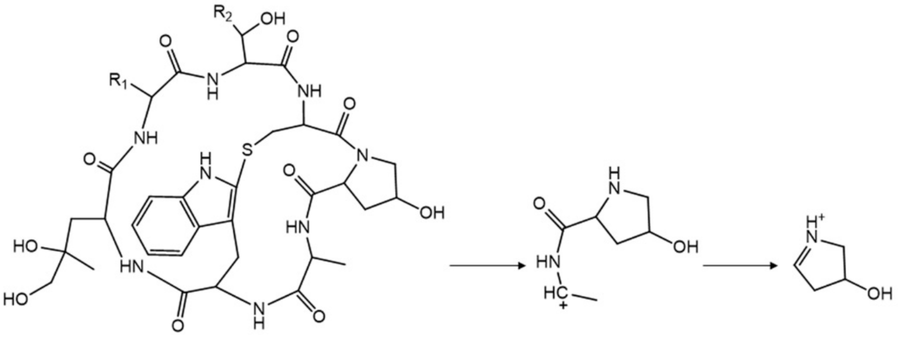

Phalloidin: $R_1$ = $CH_3$, $R_2$ = $CH_3$, Mw: 788.87 m/z: 157.10 m/z: 86.06
Phallacidin: $R_1$ = $CH(CH_3)_2$, $R_2$ = COOH, Mw: 846.90 m/z: 157.10 m/z: 86.06

**Figure S1.** The proposed fragmentation of the analytes by MS/MS mode to their products

**Table S1.** The information of 24 urine samples with mushroom poisoning

| Case | Gender | Age (year) | Time post ingestion (h) | Symptom | Intoxicated mushroom | Toxins analysis | Sample |
|---|---|---|---|---|---|---|---|
| 1 | F | 38 | 74.5 | Nausea, vomiting, abdominal pain, and watery diarrhea | Unknown | ND | 1 |
| 2 | F | 57 | 72 | Nausea, vomiting, diarrhea and palpitation | Unknown | ND | 2 |
| | | | 80 | | | ND | 3 |
| 3 | M | 61 | 96 | Nausea, vomiting and diarrhea | Unknown | ND | 4 |
| | | | 116 | | | ND | 5 |
| 4 | M | 48 | 48 | Abdominal pain, musculer pain | *Russula nigricans* and *Russula griseocarnea* | ND | 6 |
| | | | 72 | | | ND | 7 |
| 7 | M | 52 | Unknown | Nausea, vomiting and diarrhea | Unknown | ND | 8 |
| 8 | F | 51 | Unknown | Nausea, vomiting and diarrhea | Unknown | ND | 9 |
| 9 | M | 70 | 96.5 | Nausea, vomiting and palpitation | Unknown | ND | 10 |
| 10 | M | 55 | 102 | Nausea, vomiting and palpitation | Unknown | ND | 11 |

**Table S1.** *Cont.*

| | | | | | | | |
|---|---|---|---|---|---|---|---|
| 11 | F | 59 | 144 | Nausea, vomiting and skin rush | Unknown | ND | 12 |
| 12 | M | 12 | 96 | Vomiting, abdominal pain, and watery diarrhea | Unknown | ND | 13 |
| 13 | M | 36 | 96 | Vomiting, abdominal pain, and watery diarrhea | Unknown | ND | 14 |
| 14 | M | 35 | Unknown | Dizziness, nausea and diarrhea | Unknown | ND | 15 |
| 15 | M | 43 | 72 | Vomiting and diarrhea | Unknown | ND | 16 |
| 16 | M | 11 | 74 | Vomiting and diarrhea | Unknown | ND | 17 |
| 17 | M | 59 | 77 | Vomiting and diarrhea | Unknown | ND | 18 |
| 18 | M | 49 | 74 | Nausea, vomiting and diarrhea | *Amanita exitialis* | + | 19 |
| 19 | M | 59 | 74 | Nausea, vomiting and diarrhea | *Amanita exitialis* | + | 20 |
| 20 | F | 44 | 72 | Diarrhea and palpitation | Unknown | ND | 21 |
| | | | 80 | | | ND | 22 |
| 21 | M | 34 | 192 | Nausea and diarrhea | Unknown | ND | 23 |
| 22 | F | 53 | 72 | Nausea and diarrhea | *Russula virescens* | ND | 24 |

M: male; F: female; ND: not detected (< LOD); +: positive.
